# Trans-heterozygosity for mutations enhances the risk of recurrent/chronic pancreatitis in patients with Cystic Fibrosis

**DOI:** 10.1186/s10020-018-0041-6

**Published:** 2018-07-27

**Authors:** Valentina Maria Sofia, Cecilia Surace, Vito Terlizzi, Letizia Da Sacco, Federico Alghisi, Antonella Angiolillo, Cesare Braggion, Natalia Cirilli, Carla Colombo, Antonella Di Lullo, Rita Padoan, Serena Quattrucci, Valeria Raia, Giuseppe Tuccio, Federica Zarrilli, Anna Cristina Tomaiuolo, Antonio Novelli, Vincenzina Lucidi, Marco Lucarelli, Giuseppe Castaldo, Adriano Angioni

**Affiliations:** 1 Laboratory of Medical Genetics Unit, “Bambino Gesù” Children’s Hospital, IRCCS, Viale di San Paolo 15, 00146 Rome, Italy; 2 Department of Pediatrics, Tuscany Regional Centre for Cystic Fibrosis, Anna Meyer Children’s Hospital, Florence, Italy; 3 Multifactorial Diseases and Complex Phenotypes Research Area, “Bambino Gesù” Children’s Hospital, IRCCS, Rome, Italy; 4 Cystic Fibrosis Unit, “Bambino Gesù” Children’s Hospital, IRCCS, Rome, Italy; 5 Department of Medicine and Health Sciences “Vincenzo Tiberio”, University of Molise, Campobasso, Italy; 6 Regional Cystic Fibrosis Centre, United Hospitals, Mother – Child Department, Ancona, Italy; 7 Cystic Fibrosis Regional Centre (Lombardia), IRCCS Ca’ Granda Foundation, University of Milan, Milan, Italy; 8 CEINGE-Biotecnologie Avanzate, Naples, Italy; 9 Department of Neuroscience, ORL Section, University of Naples Federico II, Naples, Italy; 10 Cystic Fibrosis Support Centre, Pediatric Department, Children’s Hospital, ASST Spedali Civili, Brescia, Italy; 11 Cystic Fibrosis Regional Centre (Lazio), Sapienza University and Policlinico Umberto I, Rome, Italy; 12 Cystic Fibrosis Regional Centre (Campania), Department of Medical Transalational Sciences, Section of Pediatrics, University of Naples Federico II, Naples, Italy; 13 Cystic Fibrosis Regional Centre, Soverato Hospital, Catanzaro, Italy; 14 Department of Biosciences and Territory, University of Molise, Isernia, Italy; 15 Department of Cellular Biotechnologies and Hematology, Sapienza University of Rome, Rome, Italy; 16 Pasteur Institute, Cenci Bolognetti Foundation, Sapienza University of Rome, Rome, Italy; 17 Department of Molecular Medicine and Biotechnologies, University of Naples Federico II, Naples, Italy

**Keywords:** Cystic fibrosis, Recurrent/chronic pancreatitis, *CFTR* gene, Trypsin, Pancreatic pathways, Trans-heterozogosity

## Abstract

**Background:**

Recurrent (RP) and chronic pancreatitis (CP) may complicate Cystic Fibrosis (CF). It is still unknown if mutations in genes involved in the intrapancreatic activation of trypsin (IPAT) or in the pancreatic secretion pathway (PSP) may enhance the risk for RP/CP in patients with CF.

**Methods:**

We enrolled: 48 patients affected by CF complicated by RP/CP and, as controls 35 patients with CF without pancreatitis and 80 unrelated healthy subjects. We tested a panel of 8 genes involved in the IPAT, i.e. *PRSS1, PRSS2, SPINK1, CTRC, CASR, CFTR, CTSB* and *KRT8* and 23 additional genes implicated in the PSP.

**Results:**

We found 14/48 patients (29.2%) with mutations in genes involved in IPAT in the group of CF patients with RP/CP, while mutations in such genes were found in 2/35 (5.7%) patients with CF without pancreatitis and in 3/80 (3.8%) healthy subjects (*p* < 0.001). Thus, we found mutations in 12 genes of the PSP in 11/48 (22.9%) patients with CF and RP/CP. Overall, 19/48 (39.6%) patients with CF and RP/CP showed one or more mutations in the genes involved in the IPAT and in the PSP while such figure was 4/35 (11.4%) for patients with CF without pancreatitis and 11/80 (13.7%) for healthy controls (*p* < 0.001).

**Conclusions:**

The trans-heterozygous association between *CFTR* mutations in genes involved in the pathways of pancreatic enzyme activation and the pancreatic secretion may be risk factors for the development of recurrent or chronic pancreatitis in patients with CF.

**Electronic supplementary material:**

The online version of this article (10.1186/s10020-018-0041-6) contains supplementary material, which is available to authorized users.

## Background

Cystic Fibrosis (CF) is the most common inherited autosomal recessive disease in Caucasians. It is caused by defects in the CF *transmembrane conductance regulator* (*CFTR*) gene, which encodes a cAMP-regulated chloride channel. Defects in the CFTR protein cause abnormal chloride transport across the apical membranes of epithelial cells in the airways, pancreas, intestine, vas deferens, and sweat glands leading to progressive lung disease, pancreatic dysfunction, male infertility, and elevated sweat electrolytes, respectively (Castellani & Assael, 2017). About 85% of individuals affected by CF suffer from pancreatic insufficiency (PI), in most cases since the birth. However, 15% of the patients retain pancreatic sufficiency (PS) that permits adequate digestion (Walkowiak et al., 2008). About 85% of individuals affected by CF suffer from pancreatic insufficiency (PI), in most cases since the birth. However, 15% of the patients retain pancreatic sufficiency (PS) that permits adequate digestion (Walkowiak et al., 2008).

Recurrent pancreatitis (RP) and chronic pancreatitis (CP) may complicate CF. It was firstly mentioned by Shwachman et al. in 1975 (Shwachman et al., 1975). They reported, in a period of 20 years in 2000 patients with CF, 10 cases of pancreatitis (0.5%), all with PS. Currently, a frequency of recurrent/chronic pancreatitis between 17% (Durno et al., 2002) and 22% (Ooi et al., 2011) is estimated in patients with CF.

The dysfunction of the CFTR protein has a role in the pathogenesis of pancreatitis because it causes the impaired secretory function of pancreatic duct cells and the altered flow of digestive pro-enzymes into the duodenum triggering recurrent episodes of pancreatitis that in some patients may evolve to chronic pancreatitis (Walkowiak et al., 2008; Lew et al., 2017). This complication is more frequent in patients with CF and PS (that frequently have at least one class IV-V CFTR mutation), in which pancreatic acinar islets still produce pancreatic enzymes that may be prematurely activated within the pancreas. Recurrent/chronic pancreatitis has been observed also independently by the development of CF. In fact, Bishop et al. showed a frequency up to 30% of RP/CP in subjects carrying only one *CFTR* mutation in the absence of any sign of CF (Bishop et al., 2005). However, only a small percentage of patients with *CFTR* mutations or with CF experience RP or CP, suggesting that other risk factors must be involved (Walkowiak et al., 2008).

In fact, in addition to *CFTR*, patients with idiopathic recurrent or chronic pancreatitis have been investigated for other genes related to the premature intra-pancreatic activation of trypsin pathway. The first gene related to pancreatitis was the cationic trypsinogen gene *protease serine 1* (*PRSS1*) in 1996: a gain of function missense mutation i.e., the R122H, was identified as a risk factor for CP (Whitcomb et al., 1996). In the following years, loss-of-function variants in the *pancreatic secretory trypsin inhibitor* (*SPINK1*) (Chen et al., 2000), *calcium-sensing receptor* (*CASR*) (Felderbauer et al., 2003) and *chymotrypsinogen C* (*CTRC*) (Szmola & Sahin-Toth, 2007) genes, firmly established the pivotal role of prematurely activated trypsin within the pancreas in the etiology of pancreatitis. Moreover, our group demonstrated that mutations in several dozens of genes bearing to six different pancreatic pathways represent risk factors for recurrent/chronic pancreatitis (Sofia et al., 2016) reinforcing the concept that trans-heterozygous mutations in different genes are involved in the pathogenesis of idiopathic pancreatitis.

Interestingly, we described trans-heterozygosity for mutations in different genes also in a patient with CF and RP that was compound heterozygous for the [delta]F508 and G91G *CFTR* mutations and had a pathogenic mutation in the *CTRC* gene (Tomaiuolo et al., 2015).

Thus, to better define the role of trans-heterozygosity for mutations in different genes as a risk factor for RP/CP in patients with CF, in this study we investigated a cohort of CF patients with RP/CP in comparison to patients with CF without pancreatitis and to healthy subjects to compare the frequency of mutations in a panel of genes related to the intra-pancreatic activation of trypsin (IPAT) and a group of other genes related to pancreatic secretion pathways (PSP) previously reported to contribute to to the pathogenesis of pancreatitis (Sofia et al., 2016).

## Methods

### Patients

The informed consent was obtained from all patients or from the parents or guardians of minors. The study was approved by the Ethical Committee (Scientific Board of “Bambino Gesù” Children’s Hospital, IRCCS, Rome, Italy) and was conducted in accordance with the Helsinki Declaration.

We enrolled 48 unselected patients affected by CF complicated by RP or CP recruited through a multicentric study involving 9 Italian CF centres. The main data (i.e., age at diagnosis of CF, age at diagnosis of RP/CP, *CFTR* genotype and pancreatic status) are reported in Table 2. As control populations, we studied 35 unselected patients with CF without symptoms or history of pancreatitis (see Table 3 for the data of age at diagnosis of CF, *CFTR* genotype and pancreatic status) and 80 unrelated, adult healthy subjects of the same ethnic group of the patients with CF (i.e., Italian from at least two generations) whose DNA samples and anonymized clinical data (in particular absence of CF and of any pancreatic disorder) were available, in the biological bank of our Institution.

The diagnosis of CF was done according to the international criteria (Farrell et al., 2017). Pancreatic sufficiency in patients with CF was defined on the basis of two values of faecal pancreatic elastase > 200 mg/g measured in subjects free from acute gastrointestinal events (Walkowiak et al., 2016) or on the basis of normal 72-h fecal fat balance (Walkowiak et al., 2008). Recurrent pancreatitis was diagnosed in patients that had at least two episodes of acute pancreatitis (at a distance of at least six months after the resolution of the previous episode) each one with abdominal pain (once excluded other causes) in association with the increase of serum lipase (at least 2X the upper reference limit) and/or imaging evidence (e.g., pancreatic edema, hemorrhage or necrosis) (Morinville et al., 2012; Kumar et al., 2016). Chronic pancreatitis was diagnosed according to the M-ANNHEIM criteria (Schneider et al., 2007) in patients in which instrumental analysis revealed calcifications or characteristic ductal changes. All the patients with CP had a positive anamnesis for episodes of recurrent pancreatitis.

### Next-generation targeted sequencing of pancreatic genes

Targeted resequencing was performed using a uniquely customized design TruSeq Custom Amplicon Low Input technology (Illumina, San Diego, CA) with the MiSeq sequencing platform (Illumina). This technology is a fully integrated DNA-to-data solution, including online probe design and ordering through the Illumina website, sequencing assay, automated data analysis, and offline software for reviewing results. Online probe design was performed by entering target genomic regions into Design Studio software (Illumina). We designed a panel of eight genes included in IPAT genes (Sofia et al., 2016; Chen & Férec, 2009; Mahurkar et al., 2006; Cavestro et al., 2003): *CFTR* (NM_000492.3), *SPINK1* (NM_003122.3), *PRSS1* (NM_002769.4), *protease, serine 2* (*PRSS2*) (NM_002770.2), *CTRC* (NM_007272.2), *CASR* (NM_001178065.1), *cathepsin B* (*CTSB*) (NM_147780.2) and *keratin 8* (*KRT8*) (NM_002273). The sequence of these genes was obtained consulting the University of California, Santa Cruz, Genome Browser Home (https://genome.ucsc.edu/cgi-bin/hgGateway, last accessed October 2015) with a coverage of 100%. MiSeq system provides fully integrated on-instrument data analysis software. Each single variant reported in the vCard output file was evaluated for the coverage and the Q score and visualized via Integrative Genomics Viewer (Thorvaldosdottir et al., 2013; Robinson et al., 2011). All mutations identified by MiSeq Reporter were validated by Sanger sequencing using standard protocols.

In the second step, we selected the genes encoding proteins related to the pancreatic activation of zymogens (Sofia et al., 2016). Such genes were selected among the genes annotated in the “Pancreatic Secretion Pathway” (map04972), available in the KEGG database (Kanehisa et al., 2014). The 23 genes selected were classified into four groups according to the activity of the encoded protein or their role in the pathogenesis of pancreatitis: (i) genes encoding proteins involved in pancreatic secretion and ion homeostasis (*PPY*, *F2RL1*, *TMPRSS15*, *SCL4A2*, *SLC4A4*, *SLC26A3*, *CPB1*, *CLPS*) (Berni Canani et al., 2010; Sharma et al., 2005; Stevens et al., 2004; Multigner et al., 1985); (ii) genes encoding proteins involved in calcium (Ca^2+^) signalling and zymogen granules exocytosis (*PRKCD*, *ITPR3*, *GP2*, *TRPC3*, *STIM1*, *ATP2C2*, *TRPV1*, *TRPV5*, *TRPV6*, *PIK3CG* (Jin et al., 2015; Williams, 2008; Ramnath et al., 2010; Lupia et al., 2004); (iii) genes encoding proteins involved in autophagy (*HSP90AA1*, *LAMP2*, *MAP1LC3B*) (Willemer et al., 1989; Gukovskaya & Gukovsky, 2012; Fortunato & Kroemer, 2009) and (iv) autoimmune pancreatitis-related genes (*CA4*, *ABCF1*) (Ohmuraya & Yamamura, 2008). To search mutations in such genes, we used the targeted resequencing performed by a uniquely customized design: TruSeq Custom Amplicon Low Input Kit (Illumina) with the MiSeq sequencing platform (Illumina). The probe design (locus-specific oligos) was carried out by entering the target genomic regions into Design Studio software (Illumina). The design was performed over a cumulative target region of 99.328 bp and generated a panel of 677 amplicons with a coverage of 100% of the cumulative region. Library preparation and sequencing runs have been performed according to the manufacturer’s procedure. Only the *PRSS2* gene was analyzed by Sanger sequencing because its genomic sequence was updated in the University of California Santa Cruz (UCSC) genome database after the design of the resequencing panel.

### Data and bioinformatic analysis

The MiSeq Reporter software, a data analysis software included in the MiSeq system, performs secondary analysis on the base calls and quality score (Qscore) generated by the Real-Time Analysis software during the sequencing run and provides a list of all detected variants compared with the reference genome (*Homo sapiens*, hg19, build 37.2). Each single variant reported in the output file was evaluated for the coverage and the Qscore and visualized via the Integrative Genome Viewer (Thorvaldosdottir et al., 2013). Based on the guidelines of the American College of Medical Genetics and Genomics (Rehm et al., 2013), all regions that had been sequenced with a sequencing depth < 30 were considered not suitable for the analysis. Furthermore, we established a minimum threshold in Qscore of 30 (base call accuracy of 99.9%). All identified variants were analyzed with bioinformatic softwares evaluating the impact of change in amino-acidic structure on protein functionality with several parameters, and we filtered all variants to retain those alterations with a high disease-causing potential. We used four tools based on different parameters: PolyPhen-2 (Adzhubei et al., 2010), Align-GVGD (Mathe et al., 2006; Hicks et al., 2011), DNA SIFT (Ng & Henikoff, 2001) and MutationTaster (Schwarz et al., 2010). To facilitate the analysis of the potential splicing mutations, we used Human Splicing Finder to predict the effects of mutations on splicing signals or motifs in any human sequence (Desmet et al., 2009). Sanger sequencing using standard protocols validated the variants that have been predicted as “damaging” by at least three tools. For each of these mutations we assessed the frequency in the general population reported by the ExAC (Exome Aggregation Consortium) tool.

## Results

All individuals from the three groups, i.e., patients with CF and RP/CP (*n* = 48), patients with CF and without pancreatitis (*n* = 35) and healthy subjects (*n* = 80) were investigated for mutations in the 8 genes encoding proteins involved in IPAT and in the 23 genes encoding proteins involved in the PSP (Table 1). All the 48 patients with CF and RP/CP (Table 2) and the 35 with CF without pancreatitis (Table 3) had a pathological sweat test (i.e., > 60 mEq/L) with the exception of a patient with CF without pancreatitis that had a value of 53 mEq/L, and all patients from both the groups had two *CFTR* mutations with the exception of a patient with CF and RP in which only one mutation was known. Among the 48 patients with CF and RP/CP we found 39 patients (81.2%) with PS and 9 patients with PI (18.8%); these figures were 13/33 (30.4%) and 20 (69.6%), respectively, among the patients with CF without pancreatitis (*p* < 0.001).

**Table 1:** Number and % of subjects with mutations in IPAT genes; PSP genes and at least one gene (IPAT & PSP) in: patients with CF and recurrent/chronic pancreatitis (RP/CP); patients with CF without pancreatitis and healthy subjects

	n of cases	IPAT	PSP	IPAT & PSP
CF and RP/CP	48	14 (29.2)	11 (22.9)	19 (39.6)
CF without pancreatitis	35	2 (5.7)	3 (8.5)	4 (11.4)
healthy subjects	80	3 (3.8)	8 (10)	11 (13.7)
Chi square and (p)		20.4 (*p* < 0.001)	4.39 (*p* = 0.11)	14.5 (*p* < 0.001)

**Table 2:** Sweat chloride (mmol/L, SC), pancreatic status, age at CF and pancreatitis diagnosis, *CFTR* genotype and mutations in genes related to intra-pancreatic activation of trypsin (IPAT) and pancreatic secretion pathway (PSP) genes in 48 patients with CF and recurrent/chronic pancreatitis

ID	SC	Pancreatic status	Diagnosis of CF (Age)	RP/CP onset	*CFTR* genotype	IPAT genes	PSP genes
1	117	S	19 Y	5 Y	[delta]F508/c.2657 + 5G > A	/	/
2	77	S	20 Y	35 Y	N1303 K/P205S	/	/
3	90	S	5 M	10 Y	G85E/c.489 + 1G > T	/	*TRPV1*: c.755C > T (P252L)
4	109	S	3 M	17 Y	G542X/c.2657 + 5G > A	/	*SLC4A2*: c.299G > T (R109L); *TRPV6*: c.806C > T (T269 M)
5	77	I	2 M	8 M	[delta]F508/I1027T	/	*PIK3CG*: c.1613C > T (P538L); *TMPRSS15*: c.935C > T (T312I)
6	84	S	14 Y	26 Y	R347P/R347P	/	/
7	100	S	2 M	4 Y	c.2657 + 5G > A/c.2657 + 5G > A	/	/
8	62	S	16 Y	16 Y	[delta]F508/D110H	/	/
9	109	S	1 M	3 Y	N1303 K/c.2657 + 5G > A	/	/
10	80	S	7 M	19 Y	c.2657 + 5G > A/L1077P	/	/
11	63	S	9 Y	24 Y	W1282X/R347P	/	/
12	92	S	4 M	11 Y	[delta]F508/D579G	/	/
13	66	S	1 M	3 Y	c.579 + 1G > T/D1152H	/	/
14	74	S	43 Y	10 Y	[delta]F508/D1152H	PRSS1: c.[592-11C > T;c.592-8C > T]	/
15	67	S	46 Y	12 Y	[delta]F508/D1152H	PRSS1: c.[592-11C > T;c.592-8C > T]	/
16	60	S	10 Y	2 Y	S1297 fs*5/D993G	/	/
17	90	S	1 Y	4 Y	[delta]F508/I1000_A1004del	/	/
18	85	I	5 M	3 Y	[delta]F508/G85E	CTRC: c.514A > G (K172E)	*TRPV1*: c.1261C > T (R421X)•
19	106	S	12 Y	8 Y	N1303 K/D579G	/	/
20	76	I	9 Y	18 Y	[delta]F508/I1234V	/	/
21	78	S	11 Y	9 Y	[delta]F508/G91G	CTRC: c. 703G > A (V235I)	*PRKCD*: c.1501G > T (G501 W); *MAP1LC3B*: c.73G > C (E25Q)
22	73	S	1 Y	3 Y	[delta]F508/S1255P	/	/
23	73	I	27 Y	34 Y	Q220*/(V562I;A1006E)	*PRSS2*: c.292A > T (K98X)	*SLC26A3*: c.2276C > A (P759Q)
24	88	S	1 Y	17 Y	[delta]F508/D1152H	*PRSS2*: c.689C > T (T230I)	*SLC4A4*: c.976A > G (I326V)
25	101	I	4 M	9 Y	1717-1G > A/R334W	*PRSS2*: c.571G > A (G191R)	*ATP2C2*: c.2381G > A (R794Q)
26	100	I	1 M	10 Y	1717-1G > A/R334W	/	/
27	79	S	25 Y	na	N1303 K/R334W	/	/
28	103	S	21 Y	21 Y	N1303 K/R334W	/	*LAMP2*: c.586A > T (T196S)
29	64	S	4 Y	25 Y	R553X /2789 + 5G > A	*KRT8*: c.184G > T (G62C)	/
30	93	S	2 M	6 Y	2789 + 5G > A/2789 + 5G > A	/	/
31	110	S	4 Y	4 Y	[delta]F508/2789 + 5G > A	CTRC: c.649G > A (G217S)	/
32	76	S	16 Y	na	D614G/((TG)11 T5;V562I;A1006E)	/	/
33	75	S	50 Y	na	[delta]F508/un	*CTRC: c.514A > G (K172E)*	/
34	69	I	3 M	14 Y	N1303 K/H139R	/	/
35	119	S	4 M	23 Y	N1303 K/G85E	/	/
36	64	S	17 Y	49 Y	S549R(A > C)/R334L	/	/
37	68	S	36 Y	24 Y	[delta]F508/R334L	/	/
38	73	S	3 M	14 Y	L997F/L320 V	/	*TRPV1*: c.1781C > T (A594V)
39	65	I	5 M	5 M	[delta]F508/D110H	*KRT8: c.1073C > T (A358V)*	/
40	110	S	17 Y	32 Y	[delta]F508/S945 L	/	/
41	81	S	35 Y	40 Y	[delta]F508/2789 + 5G > A	*KRT8: c.184G > T (G62C); PRSS1: c.592-24C > T*	/
42	82	S	14 Y	28 Y	R347P/R347P	/	/
43	91	S	25 Y	30 Y	[delta]F508/2789 + 5G > A	/	/
44	114	S	7 Y	23 Y	[delta]F508/2789 + 5G > A	/	/
45	116	S	1 M	19 Y	[delta]F508/3272-26A > G	/	/
46	84	S	na	43 Y	R1066H/T501I	/	/
47	76	I	3 M	na	[delta]F508/S549 N	*CASR: c.445G > A (V149I)*	*TRPV5*: c.1726G > A (A576T)
48	60	S	16 Y	30 Y	[delta]F508/E193K	*CASR: c.565A > G (N189D)*	/

All mutations in IPAT and PSP genes were heterozygous with the exception of the c.1261C > T mutation in the *TRPV1* gene (*) that was homozygous

*S* sufficiency, *I* insufficiency, *M* months, *Y* years, *na* not available, *un* unknown

**Table 3:** Sweat chloride (mmol/L, SC), genotype of *CFTR,* intra-pancreatic activation of trypsin (IPAT) and pancreatic secretion pathway (PSP) genes in patients with CF without chronic pancreatitis

N	SC	Pancreatic status	Diagnosis of CF (Age)	*CFTR* genotype	IPAT genes (mutations)	PSP genes (mutations)
1	93	S	25 Y	[delta]F508/ [delta]F508	/	/
2	98	I	14 Y	F311 L/ M348 K/ W1145X	/	*TRPV1*: c.381C > A (C127X)
3	97	S	6 Y	[delta]F508/ [delta]F508	/	/
4	87	I	6 M	[delta]F508 / c.2046_2047insA	PRSS1/PRSS2 hybrid	*ATP2C2*: c.643G > T (D215Y)
5	76	S	3 M	G542X/ N1303 K	/	/
6	73	S	33 Y	[delta]F508 / V562I/ A1006E	/	/
7	62	S	3 M	S977F/ N1303 K	/	/
8	92	S	9 Y	G85E/ R334L	/	/
9	76	I	9 M	[delta]F508/I1234V	/	/
10	85	I	0 M	N1303 K/L1077P	/	/
11	70	S	1 M	G542X/2184insA	/	/
12	53	S	3 Y	[delta]F508/P5L	/	/
13	79	I	10 Y	R347P/P5L	/	/
14	135	I	4 M	[delta]F508/2789 + 5G > A	/	/
15	99	I	10 M	[delta]F508/S549R	/	/
16	60	I	2 M	[delta]F508/991delC	/	/
17	87	I	11 Y	R709X/ L1077P	/	/
18	79	S	4 Y	[delta]F508/ I1234V		/
19	142	S	0 M	Q39X/ CFTRdele4–11	/	/
20	98	S	4 M	[delta]F508/CFTRdele2		/
21	98	I	5 Y	[delta]F508/ Q685PfsX4	CTRC: c.649G > A (G217S)	/
22	81	I	3 Y	[delta]F508/T338I	/	/
23	80	I	2 M	[delta]F508/P5L	/	/
24	65	I	2 M	G178R/ CFTRdup19	/	/
25	90	I	1 M	[delta]F508 L732X	/	/
26	78	I	2 M	[delta]F508/G542X	/	/
27	100	I	1 M	[delta]F508/2789 + 5G > A	/	/
28	61	I	1 M	[delta]F508/N1303 K	/	/
29	86	I	3 M	2789 + G > A/2789 + G > A	/	/
30	96	I	0 M	[delta]F508/N1303 K	/	/
31	100	S	1 Y	[delta]F508/E193K	/	/
32	70	I	6 M	[delta]F508/N1303 K	/	/
33	111	I	46 Y	[delta]F508/N1303 K	/	/
34	68	I	0 M	[delta]F508/4040delA	/	/
35	100	S	33 Y	[delta]F508/ [delta]F508	/	TRPV1: c.1790C > T (T597 M)

*Het* heterozygous, *na* not available

*S* sufficiency, *I* insufficienc, *na* not available. All mutations in IPAT and in PSP genes were heterozygous

**Fig. 1:**
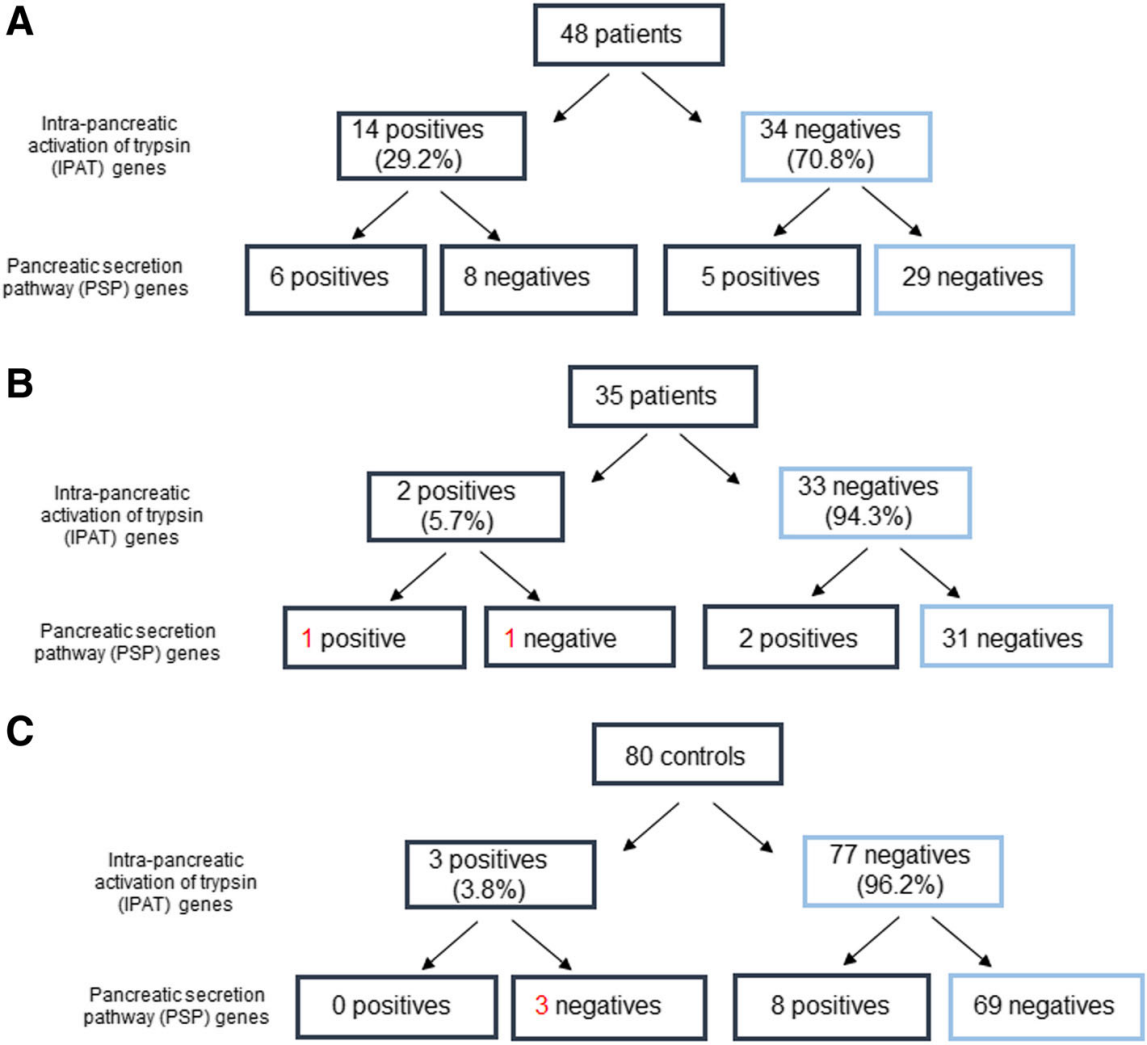
Flowchart of the results of molecular analysis in 48 patients affected by CF and recurrent/chronic pancreatitis (**a**), in 35 patients with CF and without pancreatitis (**b**) and in 80 healthy subjects (**c**)

As shown in Table 1 and Fig. 1a, in the group of patients with CF and RP/CP we found 14/48 patients (29.2%) with mutations in IPAT genes, while mutations in such genes were found in 2/35 (5.7%) patients with CF without pancreatitis (Table 1 and Fig. 1b) and in 3/80 (3.8%) healthy subjects (Table 1 and Fig. 1c) (chi square: 20.4, *p* < 0.001).

Going to the type of mutations in IPAT genes in the patients with CF and RP/CP: 2 were heterozygous for a splicing mutation in *PRSS1* in *cis*, 4 patients were heterozygous for *CTRC* mutations, 3 patients were heterozygous for *PRSS2* mutations, 2 patients were heterozygous for *CASR* mutations and 2 patients were heterozygous for *KRT8* mutations; 1 patient had a splicing mutation in *PRSS1* and a missense mutation in *KRT8* (Table 2). All these mutations were absent in our controls and were found with a frequency < 1% in the general population as annotated in the ExAC tool (Additional file 1: Table S1). While, the 2 patients with CF without pancreatitis had, in IPAT genes, a heterozygous *PRSS1/PRSS2* hybrid mutation and a heterozygous missense mutation of *CTRC*, respectively (Table 3). Finally, the three healthy subjects had all a heterozygous missense mutation in the *KRT8*, *CASR* and *SPINK1* genes, respectively (Table 4).

**Table 4:** Genotype of *CFTR* and mutations inintra-pancreatic activation of trypsin (IPAT) and pancreatic secretion pathway (PSP) genes in in healthy controls

Patient ID	IPAT genes	PSP genes
ID-823	*/*	*ITPR3:* c.2755G > T (G919C)
ID-1156	*/*	*SLC4A4:* c.2528C > T (A843V)
ID-55	*/*	*TRPV5*: c.256G > C (A86P)
ID-96	*/*	*ATP2C2*: c.629C > T (T210 M)
ID-181	*KRT8:* c.184G > T (G62C)	/
ID-182	/	*TRPV5:* c.1490 T > C (M497 T)
ID-183	/	*SLC4A4:* c.1805A > G (K602R); *TRPV5:* c.1490 T > C (M497 T)
ID-252	*CASR*: c.1672G > T (A558S)	/
ID-508	/	*ITPR3:* c.1574C > G (P525R)
ID-663	/	*ITPR3*: c.1244 T > C (L415P)
ID-352	*SPINK1:* c.101A > G (N34S)	/

All mutations in IPAT and in PSP genes were heterozygous

Thus, all individuals from the three groups were studied for mutations in the 23 genes encoding proteins of PSP. In the group of patients with CF and RP/CP (Fig. 1a, Table 1, Table 2), the analysis revealed mutations in 11/48 (22.9%) patients. While, as shown in Fig. 1b and c and in Tables 3 and 4, mutations in such genes were found in 3/35 (8.5%) patients with CF without pancreatitis and in 8/80 (10%) healthy subjects (chi square: 4.39; *p* = 0.11).

Going to the type of mutations, of the 11 patients with CF and RP/CP (Table 2) 7 patients showed heterozygous mutations, 1 displayed a homozygous mutation and 3 patients were trans-heterozygous for mutations in more than one gene. Finally (Table 2 and Additional file 1: Table S1), in this group of patients, we found 13 missense mutations in 12 genes encoding proteins of PSP: (i) *SLC4A2*, *TMPRSS15*, *SLC26A3* and *SLC4A4* genes encoding proteins involved in pancreatic secretion and ion homeostasis; (ii) *TRPV1*, *TRPV5*, *TRPV6*, *PIK3CG*, *PRKCD* and *ATP2C2* genes encoding proteins involved in calcium (Ca^2+^) signalling and zymogen granules exocytosis and (iii) *MAP1LC3B*, *LAMP2* genes encoding proteins involved in autophagy. One nonsense homozygous mutation was found in *TRPV1*. All these mutations were not present in the 35 CF patients without pancreatitis and in the 80 healthy subjects (Table 2 and Additional file 1: Table S1).

Among the 35 patients with CF without pancreatitis (Table 3), one patient was trans-heterozygous for mutations in the genes of both panels and 2 patients had mutations only in the genes of the PSP. In the cohort of healthy controls (Table 4), we found 7 individuals with variants in at least one gene of the PSP.

All the mutations found in patients and controls had a frequency < 1% in the general population (data not shown).

Finally, 19/48 (39.6%) patients with CF and RP/CP had mutations in at least one gene of the IPAT or PSP pathway. While, this is true for 4/35 (11.4%) patients with CF without CP and for 11/80 (13.7%) healthy subjects, chi square: 14.5, *p* < 0.001 (Fig. 1 and Table 1). Additional file 1: Table S1 reports a summary of all gene mutations found in the three groups of subjects studied.

## Discussion

Our study confirms that the occurrence of RP/CP is more frequent in patients with CF and PS (Walkowiak et al., 2008) and demonstrates that patients with CF and RP/CP have a significantly higher frequency of mutations in genes encoding proteins that may promote the auto-activation of pancreatic proenzymes or regulate pancreatic secretion. The small number of cases precluded clinical comparison between patients bearing mutations and those *wild-type* for all genes tested. Thus, the trans-heterozygosity for mutations in the *CFTR* and in other genes represents a risk factor for pancreatitis even in patients with CF, as we recently demonstrated for patients with idiopathic RP/CP (Sofia et al., 2016).

Among the genes encoding proteins involved in the premature activation of trypsin, we found mutations in 14 patients with CF and RP/CP in *PRSS1*, *PRSS2*, *CTRC*, *CASR* and *KRT8* genes. Eight of such mutations were known as pathogenic, while for other 6 mutations, three bioinformatic tools predicted a pathogenic effect and the ExAC tool reported the absence or the very low frequency in the general population. Going in detail, we found two mutations in *PRSS1*: the first is the [c.592-11C > T;c.592-8C > T] complex allele was found in two siblings. Keiles et al. (Keiles & Kammesheidt, 2006) described the same complex allele in an 18-years old woman with pancreatitis; she also carried the T908 N *CFTR* mutation. Furthermore, the *PRSS1* splicing mutation c.592-24C > T previously described in two siblings with CP (Singhi et al., 2014) was found in a patient with the *CFTR* genotype [delta]F508/2789 + 5G > A. The patient carried also a *KRT8* mutation.

Thus, we found three mutations in *PRSS2*. The T230I and K98X mutations are novel. The T230I was reported as pathogenic by the three bioinformatic tools; the K98X is a nonsense mutation causing an early stop codon. The third mutation in *PRSS2* gene, i.e., the G191R, had been analysed by Witt et al. (Witt et al., 2006). They demonstrated that the recombinant G191R protein showed a complete loss of trypsin activity owing to the introduction of a new tryptic cleavage site rendering the enzyme hypersensitive to autocatalytic proteolysis. Furthermore, we found three missense mutations in *CTRC*. The K172E was identified in two patients. Masson et al. described the K172E mutation in a patient with idiopathic chronic pancreatitis (Masson et al., 2008). Thus, in two other patients with the *CFTR* [delta]F508/G91G and [delta]F508/2789 + 5G > A genotype we identified the *CTRC* V235I and G217S missense mutations, respectively. Rosendahl et al. investigated the functional consequences of these two *CTRC* missense mutations through transient transfections in HEK 293 T cells (Rosendahl et al., 2008). They demonstrated that the G217S causes a loss-of-function of the *CTRC* protein, whereas the V235I results in normal or slightly reduced function, respectively. Moreover, data observed in another report suggest a role for the V235I mutation in triggering the pancreatic phenotype in a patient with CF (Tomaiuolo et al., 2015). Rosendahl identified the G217S mutation also in a healthy control and similarly, in our study we found it in a patient with CF without RP/CP thus, we cannot conclude on the pathogenic role of such mutation.

In addition, we found two missense mutations in *CASR*: the V149I and the N189D; both the mutations were considered pathogenic by bioinformatic tools and by the very low frequency in the general population.

Finally, we found two missense mutations in *KRT8*. The G62C was identified in two PS patients: the first case had the R553X/2789 + 5G > A *CFTR* genotype and the second had the [delta]F508/2789 + 5G > A *CFTR* genotype in addition to the c.592-24C > T mutation in *PRSS1.* Initially, the *KRT8* G62C mutation was considered pathogenic by Cavestro and coworkers (Cavestro et al., 2003). Later, Witt et al. observed that the frequency of the mutation did not differ between patients with acute or chronic pancreatitis, pancreatic adenocarcinoma and control individuals (Witt et al., 2006). Also in our study, this mutation was found in a healthy subject. Thus, we cannot conclude on the pathogenic role of such mutation. Finally, the *KRT8* A358V novel mutation was identified in a patient with the [delta]F508/D110H *CFTR* genotype and we speculate on its pathogenetic role on the basis of the bioinformatic prediction and of its absence in alleles from the general population. Interestingly, in a recent study it was demonstrated an interaction between KRT8 and the CFTR protein that could influence the function of CFTR (Treiber et al., 2006).

All the genes discussed so far are involved in the premature intra-pancreatic activation of trypsin. This pathway plays a pivotal role in triggering the activation cascade of all pancreatic digestive zymogens caused by the breaking of the interactions of these proteins in pancreas leading to injury of acinar cells and consequently recurrent attacks of pancreatitis (Sofia et al., 2016; Chen & Férec, 2009).

Moving to patients with CF without pancreatitis, we found two cases with mutations IPAT genes: a patient had the *PRSS1*/*PRSS2* hybrid and a the *CTRC* G217S, a mutation previously identified in a normal subject by Rosendahl et al. (Rosendahl et al., 2008). In healthy subjects, we identified three individuals with mutations in *SPINK1*, *CASR* and *KRT8* genes. The N34S mutation identified in *SPINK1* was described by Threadgold et al. as a variation associated with a familial pattern of idiopathic chronic pancreatitis (Threadgold et al., 2002). Actually, it is considered not disease causing being found in normal control too with an average prevalence of 2.5% and an allele frequency of 1.25% (Premchandar & s, 2017). The missense A558S identified in *CASR* is a novel mutation considered potentially pathogenic by bioinformatic analysis despite a frequency of about 1% in the general population, while the *KRT8* G62C mutation found in another healthy control was identified also in healthy subjects by Witt et al. (Witt et al., 2006).

All mutations found in the second group of 23 genes PSP in the three groups of subjects were classified as possibly damaging by the three bioinformatic tools and all but two had a frequency in the general population < 0.1%. However, even if there is a trend of higher frequency of such mutations in patients with CF and RP/CP as compared to the patients with CF without pancreatitis and to healthy controls, the difference is not significant. To be noted that 6 out of 14 patients affected by CF with RP/CP with mutations in genes encoding proteins potentially involved in premature intra-pancreatic activation of trypsin also have mutations in genes of the pancreatic secretion pathway, in particular those belonging to the Ca^2+^ signalling, pancreatic secretion and autophagy pathways, further reinforcing the concept that trans-heterozygous mutations in different genes may have a synergic effect in the pathogenesis of RP/CP.

## Conclusions

Our data strongly suggest that the trans-heterozygosity for mutations in CFTR and in genes encoding proteins involved in IPAT and PSP may enhance the risk for RP/CP in patients with CF, as we previously demonstrated in subjects with idiopathic RP/CP (Sofia et al., 2016). Further studies are called, to define if patients with trans-heteroygous mutations have a more severe outcome of pancreatitis (the small number of cases limited such evaluation in the present study) and functional studies are necessary to elucidate the pathogenetic mechanism of pancreatitis in patients bearing mutated genes/proteins.

## Additional file

Additional file 1: **Table S1.** List of mutations in genes involved in the intrapancreatic activation of trypsin (IPAT) and pancreatic secretion pathway (PSP) and allelic frequency (%) in the three groups of subjects studied (A: CF with RP; B: CF without RP; C: healthy controls) and D: in the general population (ExAC tool). (DOCX 17 kb)

## Abbreviations

CF: cystic fibrosis
CFTR: cystic fibrosis transmembrane conductance regulator
CP: chronic pancreatitis
IPAT: intra-pancreatic activation of trypsin
PI: pancreatic insufficiency
PS: pancreatic sufficiency
PSP: pancreatic secretion pathways
RP: recurrent pancreatitis
