# Supplementary material for: Trans-heterozygosity for mutations enhances the risk of recurrent/chronic pancreatitis in patients with Cystic Fibrosis
Source: Mol Med. 2018 Jul 27;24:38. doi: 10.1186/s10020-018-0041-6 (PMC6062922; doi:10.1186/s10020-018-0041-6)
Supplement: Supplementary file 1 — Table S1. List of mutations in genes involved in the intrapancreatic activation of trypsin (IPAT) and pancreatic secretion pathway (PSP) and allelic frequency (%) in the three groups of subjects studied (A: CF with RP; B: CF without RP; C: healthy controls) and D: in the general population (ExAC tool). (DOCX 17 kb) [file 10020_2018_41_MOESM1_ESM.docx]

**Table S1**: list of mutations in genes involved in the intrapancreatic activation of trypsin (IPAT) and pancreatic secretion pathway (PSP) and allelic frequency (%) in the three groups of subjects studied (A: CF with RP; B: CF without RP; C: healthy controls) and D: in the general population (ExAC tool).

**IPAT GENES**

***SPINK1*** A B C D

c.101A>G (N34S) 0 1 0 0.98

***PRSS1***

c.[592-11C>T;c.592-8C>T] 2 0 0 na

c.592-24C>T 1 0 0 na

***PRSS2***

c.292A>T (K98X) 1 0 0 na

c.689C>T (T230I) 1 0 0 na

c.571G>A (G191R) 1 0 0 na

**PRSS1/PRSS2 hybrid**  0 1 0 0

***CTRC***

c.514A>G (K172E) 2 0 0 0.88

c.649G>A (G217S) 1 1 0 0.01

c.703G>A (V235I) 1 0 0 0.12

***CASR***

c.445G>A (V149I) 1 0 0 0

c.565A>G (N189D) 1 0 0 0

c.1672G>T (A558S) 0 1 0 0

***KRT8***

c.184G>T (G62C) 2 0 1 0.5

c.1073C>T (A358V) 1 0 0 0

***CTSB*** (none mutation)

**PSP GENES**

***TMPRSS15***

c.935C>T (T312I) 1 0 0 0.13

***SCL4A2***

c.299G>T (R109L) 1 0 0 0.01

***SLC4A4***

c.976A>G (I326V) 1 0 0 0

c.2528C>T (A834V) 0 0 1 0

c.1805A>G (K602R) 0 0 1 na

***SLC26A3***

c.2276C>A (P759Q) 1 0 0 0

***TRPV1***

c.755C>T (P252L) 1 0 0 0

c.1261C>T (R421X) 1 0 0 0.01

c.1782C>T (A594V) 1 0 0 0.01

c.381C>A (C127X) 0 1 0 0

c.1790C>T (T597M) 0 1 0 0

***TRPV5***

c.256G>C (A86P) 0 0 1 0

c.1726G>A (A576T) 1 0 0 0.02

c.1490T>C (M497T) 0 0 2 0

***TRPV6***

c.806C>T (T269M) 1 0 0 0.07

***PIK3CG***

c.1613C>T (P538L) 1 0 0 0.07

***PRKCD***

c.1501G>T (G501W) 1 0 0 0

***ATP2C2***

c.629C>T (T210M) 0 0 1 0.01

c.2381G>A (R794Q) 1 0 0 na

c.643G>T (D215Y) 1 0 0 0.04

***MAP1LC3B***

c.73G>C (E25Q) 1 0 0 0.64

***LAMP2***

c.586A>T (T196S) 1 0 0 0.02

***ITPR3***

c.1574C>G (P525R) 0 0 1 0

c.2755G>T (G919C) 0 0 1 0

c.1244T>C (L415P) 0 0 1 0

***PPY*** (none mutation)

***F2RL1*** (none mutation)

***CPB1*** (none mutation)

***CLPS*** (none mutation)

***GP2*** (none mutation)

***TRPC3*** (none mutation)

***STIM1*** (none mutation)

***HSP90AA1*** (none mutation)

***CA4*** (none mutation)

***ABCF1*** (none mutation)
